# Overexpression of Heme Oxygenase-1 in Mesenchymal Stem Cells Augments Their Protection on Retinal Cells In Vitro and Attenuates Retinal Ischemia/Reperfusion Injury In Vivo against Oxidative Stress

**DOI:** 10.1155/2017/4985323

**Published:** 2017-02-01

**Authors:** Li Li, GaiPing Du, DaJiang Wang, Jin Zhou, Guomin Jiang, Hua Jiang

**Affiliations:** ^1^Department of Ophthalmology, Jinan Military General Hospital, No. 25 Shifan Road, Tianqiao District, Jinan 250031, China; ^2^Department of Ophthalmology, The 88th Hospital of Chinese People's Liberation Army, No. 6 Hushan East Road, Tai'an 271000, China; ^3^Department of Ophthalmology, 401 Hospital of Chinese People's Liberation Army, No. 22 Minjiang Road, Qingdao 266071, China; ^4^Department of Ophthalmology, Chinese People's Liberation Army General Hospital, No. 28 Fuxing Road, Haidian District, Beijing 100853, China; ^5^Department of Cardre Ward, The 316th Hospital of Chinese People's Liberation Army, No. A2 Niangniangfu, Xiangshan Road, Haidian District, Beijing 100093, China; ^6^Department of Ophthalmology & Visual Science, University of Louisville, 301 E. Muhammad Ali Blvd., Louisville, KY 40202, USA

## Abstract

Retinal ischemia/reperfusion (I/R) injury, involving several ocular diseases, seriously threatens human ocular health, mainly treated by attenuating I/R-induced oxidative stress. Currently, mesenchymal stem cells (MSCs) could restore I/R-injured retina through paracrine secretion. Additionally, heme oxygenase-1 (HO-1) could ameliorate oxidative stress and thus retinal apoptosis, but the expression of HO-1 in MSC is limited. Here, we hypothesized that overexpression of HO-1 in MSC (MSC-HO-1) may significantly improve their retina-protective potentials. The overexpression of HO-1 in MSC was achieved by lentivirus transduction. Then, MSC or MSC-HO-1 was cocultured with retinal ganglion cells (RGC-5) in H_2_O_2_-simulated oxidative condition and their protection on RGC-5 was systemically valuated in vitro. Compared with MSC, MSC-HO-1 significantly attenuated H_2_O_2_-induced injury of RGC-5, including decrease in cellular ROS level and apoptosis, activation of antiapoptotic proteins p-Akt and Bcl-2, and blockage of proapoptotic proteins cleaved caspase 3 and Bax. In retinal I/R rats model, compared with control MSC, MSC-HO-1-treated retina significantly retrieved its structural thickness, reduced cell apoptosis, markedly attenuated retinal oxidative stress level, and largely regained the activities of typical antioxidant enzymes, SOD and CAT. Therefore, it could be concluded that overexpression of HO-1 provides a promising strategy to enhance the MSC-based therapy for I/R-related retinal injury.

## 1. Introduction

Retinal ischemia/reperfusion (I/R) injury plays a pivotal role in the pathogenesis of a series of ocular diseases, including diabetic retinopathy, acute glaucoma, and retinopathy of prematurity. Usually, I/R injury may result in vision-loss and even blindness due to permanent damage to the retina, especially retinal neurons [1–3]. Retinal I/R generally consisted of two courses, the ischemia and the successive reperfusion. During the ischemia status, blood flow to retina was blocked, causing the deficiency of oxygen and other nutrients and thus the depletion of adenosine triphosphate [4], while, in the course of the reperfusion, the tissue damage was aggravated due to the generation of reactive oxygen species (ROS) and proinflammatory mediators that subsequently led to oxidative stress and inflammation [5, 6]. Retinal neuronal injury is mainly ascribed to oxidative stress [7, 8] and thus antioxidative treatments were regarded as one of the main therapies for retinal I/R injury [9].

Currently, cell transplantation attracted a widespread interest in medical applications due to the production of various trophic factors in vivo via the immature cells, such as stem cells [10, 11]. Mesenchymal stem cells (MSC) are the archetype of multipotent stem cells for their abundant autologous source and delivery via an allogeneic fashion [12]. In addition, MSC were also able to secrete several cytokines and nutrients [13, 14], which can significantly reduce surrounding cellular oxidative stress and the resulting apoptosis [15, 16]. Furthermore, the developing technologies of cell culture and genetic engineering [17, 18] further promote the therapeutic application of MSC through integrating other positive treatments [19, 20], which also can overcome the possible side effects from monotherapy in clinical practice [21]. Previous study confirmed that MSC transplantation can treat retinal I/R injury by expressing neurotropic factors [22]. However, key therapeutic factors, such as HO-1, were naturally low expressed in MSC.

Heme oxygenase-1 (HO-1), an antioxidant and cytoprotective enzyme [23], is one of members of the heme oxygenase family [24, 25], which equimolarly decompose heme to biliverdin, free iron, and carbon monoxide (CO). A series of studies, including HO-1 promoter polymorphisms, HO-1 antisense, and knockout, have clarified the central role of HO-1 in intracellular antioxidant defenses [26, 27]. Its product of decomposition, biliverdin, can further be metabolically degraded into bilirubin. Both of them display a potent antioxidative capacity against intracellular oxidative stress. Moreover, bilirubin also possesses cytoprotective and anti-inflammatory capability [28, 29]. It has been evidenced that CO, another product of the HO-1 induced degradation of heme, has antiapoptotic and cytoprotective role in the process of anti-inflammatory [30]. Additionally, recent study has applied HO-1 to treat retina related diseases against oxidative stress, harvesting plausible protective effects on retinal endothelial cells [20].

Based on the capacity of HO-1 to protect retina against oxidative stress, we herein incorporated HO-1 gene into MSC through lentivirus transduction, aiming to promote the therapeutic efficiency of MSC. The feasibility was first confirmed by employing in vitro transwell indirect culture in H_2_O_2_-simulated oxidative stress medium. Subsequently, we transplanted HO-overexpressing MSC into rats with retinal post-I/R injury for practical attempts. In addition, we preliminarily studied the underlying mechanism of enhanced restoration of retina with HO-1 overexpression MSC by investigating the level of antioxidant enzyme and the expression of apoptosis-related proteins.

## 2. Materials and Methods

### 2.1. Isolation and Culture of Cells

Rat retinal ganglion cells (RGC-5, American Type Culture Collection) were cultivated with DMEM (Gibco) supplemented with 10% FCS (PAA Laboratories), 100 IU/mL penicillin, and 100 *μ*g/mL streptomycin (Sigma). RGC-5 were grown to confluency and then enzymatically dissociated with 0.2% trypsin (Invitrogen) and 0.05% EDTA (PAA Laboratories). The medium was refreshed every 3 days.

Two-week-old Sprague-Dawley rats were used for the isolation of MSC from the inguinal adipose tissue according to the previously described protocol [12]. In brief, the inguinal hair of rats was carefully shaved and the exposed inguinal skin successively underwent 5 min sterilization with 75% ethanol. Subsequently, the tissue isolation surgery was carried out under sterile condition. The obtained adipose tissue was washed thoroughly with PBS and cut into small mass. Then, the tissues were digested for 30 min with 0.1% collagenase I (Sigma) and 0.05% trypsin in serum-free *α*MEM (Gibco). The digestion was ceased by supplementing equal volume of *α*MEM containing 10% FBS (Hyclone). The mixed solution was filtered through 80 *μ*m mesh. The cell remains were further placed onto culture dishes and incubated in *α*MEM containing 10% FBS at 37°C and 5% CO_2_. The adherent cells were used as the first passage of MSC and these MSC were expanded to the 5th passage. Cells were passaged when they reached about 90% confluence and passage 3 cells were used in the in vitro and in vivo experiments. Subsequently, fluorescence-activated cell sorting (FACS) with CD29, CD90, CD45, and CD34 markers was used to verify the cellular identity of cells.

### 2.2. Multipotent Differentiation

*Osteogenic Induction*. MSC at the density of 5,000 cells/cm^2^ were induced towards osteogenic differentiation for 21 days (alpha MEM medium supplemented with 100 nM dexamethasone, 10 mM *β*-glycerophosphate, and 50 mM ascorbic acid-2-phosphate (Wako Chemicals, Richmond, VA) with 10% FBS and 1% penicillin and streptomycin). Afterwards, the osteoblasts were bathed in 95% ice-cold ethanol for 5 minutes and stained with 2% Alizarin Red Solution (pH = 4.0). Calcium deposits recognized as orange-red stained areas in cells were identified under light microscopy.

*Adipogenic Induction*. MSC at the density of 20,000 cells/cm^2^ were induced towards adipogenic differentiation for 21 days (alpha MEM medium supplemented with 10% FBS, 1% penicillin and streptomycin, 1 mM dexamethasone, 500 mM 3-isobutyl-1-methylxanthine, 10 mg/mL insulin, and 100 mM indomethacin). The induced cells were fixed with 4% paraformaldehyde for 30 min at room temperature and stained with fresh Oil Red O solution for another 50 min. The formed fat droplets were observed under bright field microscope.

### 2.3. Modification of HO-1 Gene in MSC via Lentiviral Transduction

Lentiviral vectors with or without HO-1 were labeled by GFP. First, total RNA was harvested via the usage of the PrimeScript RT reagent kit, according to the manufacturer's instructions (TAKARA-BIO, Shiga, Japan). The obtained RNA was then converted into complementary DNA (cDNA) using a Reverse Transcription System Kit (Applied Biosystems, Foster City, CA, USA). The primers of HO-1 gene were synthesized using Primer Premier 5.0 software, based on its cDNA sequences from the GenBank database (GenBank, Accession: NM_012580.2). The PCR-amplified gene was inserted into the LV5 vector (GenePharma Co., Ltd., Shanghai, China), according to the manufacturer's protocols. The EF-1*α* promoter was used to trigger gene expression. After transfecting into 293 T cells for 3 days, the viruses were collected and concentrated by ultracentrifugation.

Lentivirus transductions without (MSC) or with HO-1 gene (MSC-HO-1) were conducted according to the previously established method with small modifications. Briefly, MSC were incubated with lentivirus at the MOI of 20 for 24 h at 37°C. Subsequently, cells were washed with PBS for several times to remove redundant lentivirus. Then, the cells were incubated with fresh culture medium. Two days later, the transfection activities of MSC were investigated by the expression of GFP through a fluorescent microscope. Those uninfected cells were removed by adding puromycin (1 *μ*g/mL).

### 2.4. MSC and RGC-5 Transwell Coculture

The coculture of MSC and RGC-5 was conducted according to previously reported method [31]. In brief, MSC were seeded at 1 × 10^5^ cells/well onto 24-well transwell permeable support (pore size: 0.4 *μ*m, Corning, NY, USA), followed with overnight incubation at 37°C, 5% CO_2_. RGC at a density of 5 × 10^4^ cells/well were then seeded into the bottom of 24-well plates. After cocultivation for 24 h, 100 *μ*M of H_2_O_2_ was added in the coculture medium and cells were incubated for another 24 h.

### 2.5. Determination of Intracellular Reactive Oxygen Species Levels

For determining the level of intracellular ROS, RGC-5 were incubated with 10 *μ*M oxidation-sensitive fluorescent probe: 2′,7′-dichlorodihydrofluorescein diacetate (S carboxy-H2DCFDA) at 37°C in the darkness for 20 min. Subsequently, the cells were rinsed with 1x PBS (pH 7.4) to remove the unloaded dye. The cells were trypsinized, collected with polystyrene tubes with cell-strainer caps (Falcon), and examined with flow cytometry. Geometric distributions of side-angle light scatter height (SSC-H) and carboxyl-DCF fluorescence were obtained and analyzed using FACS (FACScan, Becton Dickinson) and Cell Quest 3.2 (Becton Dickinson) software.

### 2.6. Retinal Ischemia/Reperfusion Injury

Sprague-Dawley rats with similar body weights were first anesthetized using sodium pentobarbital and underwent surgery according to previous description [32]. Briefly, a 27-gauge infusion needle linked to normal saline reservoir was cannulated into the anterior chamber of the right eye. The intraocular pressure was modulated up to 110 mmHg for 60 min through augmenting the amount of normal saline. The fundus whitening and the retinal blood flow restoration were used to confirm retinal ischemia and reperfusion. The control group was treated with Sham-procedure, maintaining normal ocular tension.

### 2.7. Cell Transplantation

Transplantation of MSC was performed according to the previous method [32]. The right eye of rat was chosen to receive injection treatment. After anesthetization, pupils of rats were dilated and the eyes were then slowly protruded with a rubber sleeve. A 90° peritomy was conducted in the temporal quadrant followed by a sclerotomy at ~1 mm behind the limbus with a 27-gauge needle. Afterwards, a 33-gauge blunt-tip needle was tangentially inserted towards the posterior pole of the eye. 5 *μ*L MSC suspension (1 × 10^5^ cells/mL) or 5 *μ*L PBS was gently injected into the subretinal space. Rats were euthanatized and the eyes were enucleated after 7, 14, and 21 days, respectively.

### 2.8. Western Blotting Assay

Cells grown in 6-well plates were washed with PBS twice and lysed harnessing Laemmli Sample Buffer (Bio-Rad). After centrifugation at 4°C, the protein part was collected and was analyzed using BCA™ Protein Assay Kit (Pierce). Protein sample (60 *μ*g) was loaded in sodium dodecyl sulfate PAGE gel. The obtained discrete gel proteins were then electrophoretically transferred to nitrocellulose membranes, followed by incubating with primary antibodies against HO-1, p-Akt, cleaved caspase 3, Bax, or Bcl-2 overnight at 4°C. The corresponding secondary antibodies marked with HRP were incubated for 1 h at room temperature. Akt was used as the internal reference for p-Akt, while GAPDH served as internal reference for other three factors. The antibodies used in this study were all purchased from Cell Signal Technology.

### 2.9. Measurement of Retinal Thickness

The retinal thicknesses of rats were measured according to the previously described method. Briefly, the rats received surgeries under anaesthetization at day 21 and their eyes were then enucleated. After the careful removal of cornea, lens, and vitreous, the eye-cups were fixed in 4% paraformaldehyde for 2 h, immersed in 30% sucrose solution overnight at 4°C, and then embedded in OCT media (Sakura Finetek). Retinal sections of 0.5 mm, attained by cutting along the vertical meridian of eye and crossing the optic nerve head, were stained with hematoxylin and eosin (H&E) and tested using light microscope. Measurements were taken at every 250 *μ*m in a range of 800–1200 *μ*m centralized at the optic nerve head.

### 2.10. Terminal Deoxynucleotidyl Transferase dUTP Nick End Labeling (TUNEL) Assay for Cell Apoptosis

TUNEL was performed using the “one step cell death detection kit-fluorescein” (Beyotime Biotechnology) with fluorescein isothiocyanate (FITC) for the in vitro assay or streptavidin-tetramethylrhodamine (TRITC) for the in vivo assay, according to the manufacturer's protocols. Typically, the air-dried cells were fixed in 4% paraformaldehyde for 1 h at 20°C and permeabilized for 2 min on ice with 0.1% Triton X-100 containing 0.1% sodium citrate. The TUNEL reagent was incubated with the cells for 1 h at 37°C in the darkness. After removing unreacted compounds with 10 mM PBS, the cells were placed onto glass slides and cultured with a DAPI-containing antifade mounting medium. As for the in vivo TUNEL assay, rats were first euthanized and retinal sections were harvested as described above. TUNEL staining (green in vitro and red in vivo) was imaged and recorded via an Olympus (Tokyo, Japan) FluoView-FV300 Laser Scanning Confocal System. The cellular apoptosis was expressed as the proportion of TUNEL-positive with DAPI-positive (blue) nuclei.

### 2.11. Measurement of Reactive Oxygen Species in the Retina

The ROS levels in the rat retina were analyzed using luminol (5-amino-2,3-dihydro-1,4-phthalazinedione, Sigma) and lucigenin (bis-N-methylacridiniumnitrate, Sigma). Briefly, retinas were isolated at weeks 1, 2, and 3 after surgery and then homogenized with 10 mM PBS. Tissue samples were transferred into vials in the presence of PBS-HEPES buffer (0.5 M PBS containing 20 mM HEPES, pH 7.2). The levels of ROS were assessed by adding the chemilumigenic probes lucigenin and luminol (final concentration of 0.2 mM). The luminescent counts were recorded at 1 min intervals at room temperature via luminescence reader (BioTek). Results were given as counts per min (counts/min) for a counting period of 5 min (rlu/mg tissue).

### 2.12. Measurements of the Activities of Antioxidant Enzymes in the Retina

Superoxide dismutase (SOD) and catalase (CAT) activities in the retina were measured by colorimetric assays at weeks 1, 2, and 3. Both SOD and CAT assay kits were from Cayman Chemical (Ann Arbor, MI, USA). Assay procedures and tissue homogenate preparations were conducted according to the manufacturer's protocols.

### 2.13. Statistical Analysis

Data were expressed as mean ± standard deviation (SD). Statistical significance was set at *P* values < 0.05. The analysis among groups exploited one-way ANOVAs followed by Tukey's post hoc test for multiple pairwise examinations.

## 3. Results

### 3.1. Characteristics of MSC and HO-1 Transduction by Lentiviral Vectors

**Figure 1:**
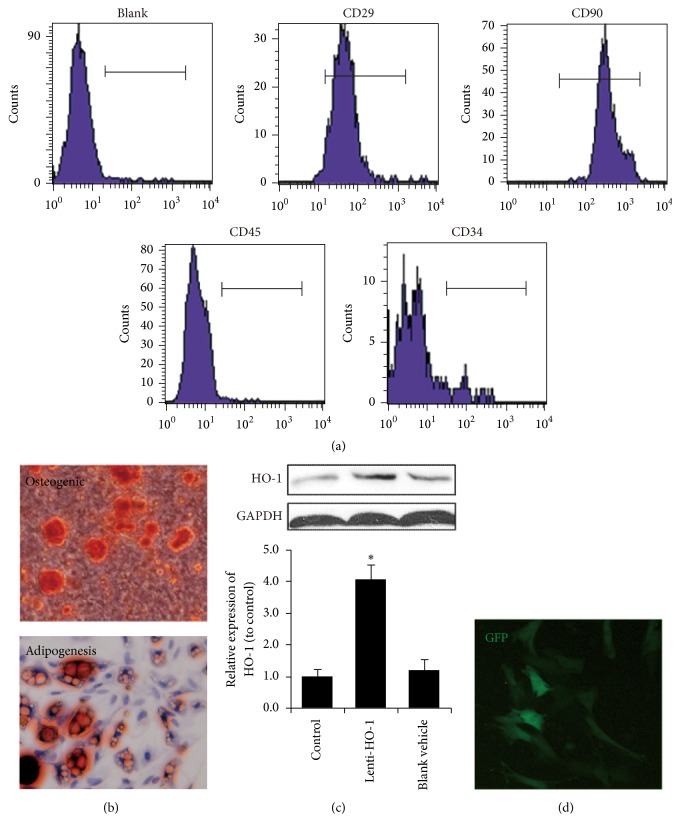
Characteristics of MSC and HO-1 transduction by lentiviral vectors. (a) Flow cytometry demonstrated that most MSC expressed CD29 and CD90 but less CD45 and CD34. (b) Multipotency of MSC, differentiating into adipocytes stained by Oil Red O and osteocytes stained by Alizarin Red S. (c) HO-1 expressions in control and MSC transduced with lenti-HO-1 and blank vehicle, *n* = 5. (d) GFP image of MSC after transduction by lentiviral vectors. ^*∗*^*P* < 0.01 compared with control.

We first systematically characterized the immune phenotype and differentiation potential of isolated cells to verify their identities. As showed in Figure 1(a), the majority of the isolated cells were positive CD29 and CD90, while they were negative for CD31 and CD45. The multipotency of MSC was analyzed by osteogenic and adipogenic differentiation. As shown in Figure 1(b) the oil droplets stained with Oil Red O and osteoblasts producing calcium stained with Alizarin Red Sin clearly demonstrated their multidifferentiation capabilities.

After lentiviral transduction, we examine the expression of HO-1. As showed in Figure 1(c), a significantly higher expression was observed only in the HO-1 gene transduction group, while there were no differences between control and blank vehicle group. GFP was used to identify grafted cells and the fluorescence of GFP was observed in MSC after lentiviral transduction (Figure 1(d)).

### 3.2. HO-1 Overexpression in MSC Protects RGC-5 against Oxidative Stress

**Figure 2:**
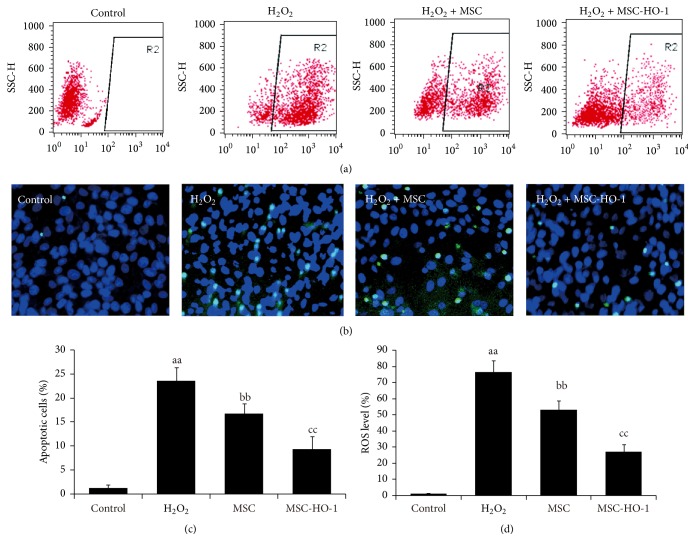
The effects of HO-1 overexpression in MSC on intracellular ROS levels and apoptosis. MSC or MSC-HO-1 were indirectly cocultivated with RGC-5 after adding H_2_O_2_. (a) Intracellular ROS levels in RGC-5 were analyzed by flow cytometry. (b) Representative TUNEL images of RGC-5. (c) and (d) The quantitative detection of ROS levels and apoptosis of RGC-5 (*n* = 5). ^aa^*P* < 0.01 compared with control; ^bb^*P* < 0.01 compared with H_2_O_2_ group; ^cc^*P* < 0.01 compared with MSC group.

To assess whether HO-1 overexpression in MSC was able to reduce apoptosis of RGC-5 against oxidative stress, H_2_O_2_ were supplemented in RGC-5 culture medium to induce intracellular oxidative stress and ROS levels were first investigated. As showed in Figure 2(a), normal cells contain a relatively lower ROS level and weak carboxyl-DCF fluorescence was detected, while the majority of cells in H_2_O_2_ treated group were detected with high-ROS level. After introducing MSC, the augment of high-ROS cells produced by H_2_O_2_ could be offset and this effect could be further enhanced by the overexpression of HO-1 in MSC. The typical TUNEL staining imaging (Figure 2(b)) also complied with the flow cytometry results, showing that TUNEL-positive cells were much less presented in the MSC-HO-1 treated group than that in the MSC-treated one. The following quantification results (Figures 2(c) and 2(d)) showed that apoptosis in MSC-HO-1 treated group was significantly lower than MSC-treated group, as well as the intracellular ROS level.

### 3.3. Assessment of Apoptosis-Related Proteins

**Figure 3:**
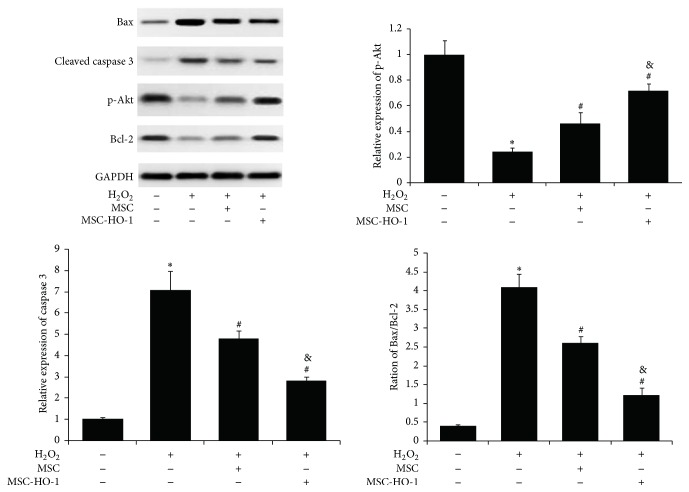
Assessment of apoptosis-related proteins in RGC-5. The levels of apoptotic proteins in H_2_O_2_ treated RGC-5 without or with MSC or MSC-HO-1, respectively, were analyzed by western blotting (*n* = 5). ^*∗*^*P* < 0.01 compared with control; ^#^*P* < 0.01 compared with H_2_O_2_ group; ^#^*P* < 0.01 compared with H_2_O_2_ + MSC group.

To further understand the underlying mechanism, we investigated the expression of apoptotic proteins in RGC-5. As shown in Figure 3, H_2_O_2_ treatment significantly upregulated proapoptotic proteins, Bax and cleaved caspase 3, and downregulated antiapoptotic proteins, p-Akt and Bcl-2. These changes in RGC-5 were significantly reversed when MSC were introduced. We also found that the MSC-based restoration could be further promoted by the overexpression of HO-1. Together, these findings demonstrated the potent protection of MSC overexpressing HO-1 on RGC-5 against oxidative stress.

### 3.4. Effects of MSC Overexpressing HO-1 on Retinal Histology after I/R Injury

**Figure 4:**
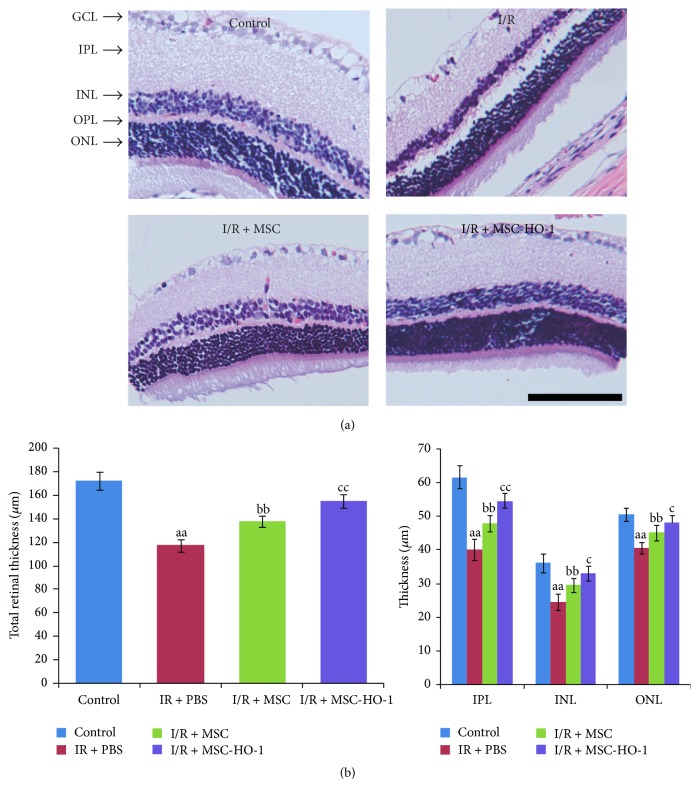
Effects of MSC-HO-1 on retinal histology at 21 days after I/R injury. (a) Representative retinal section images of control, I/R + PBS, I/R + MSC, or I/R + MSC-HO-1 group were achieved by H&E-staining. (b) The thickness of total retina (from the inner limiting membrane to the pigment epithelium), the inner plexiform layer (IPL), the inner nuclear layer (INL), and the outer nuclear layers (ONL) was investigated in the eyes of control, I/R + PBS, I/R + MSC, or I/R + MSC-HO-1 group, respectively (*n* = 5). ^aa^*P* < 0.01 compared with control group; ^bb^*P* < 0.01 compared with IR + PBS group; ^cc^*P* < 0.01 compared with IR + MSC group; ^c^*P* < 0.05 compared with IR + MSC group.

As showed in Figure 4, retinal I/R injury obviously slimed the whole retina compared with control group at day 21. There was marked restoration of the structure in the MSC group, especially in the MSC-HO-1 group, compared with the I/R group. The data of bar plotting clearly demonstrated that the significant decrease in the total retinal thickness, inner plexiform layer (IPL), inner nuclear layer (INL), and outer nuclear layer (ONL) in the eyes of I/R group was significantly attenuated by introducing MSC. Moreover, the beneficial effect of MSC could be further amplified by the overexpression of HO-1 (Figure 4).

### 3.5. Effects of MSC-HO-1 Transplantation on Retinal Apoptosis

**Figure 5:**
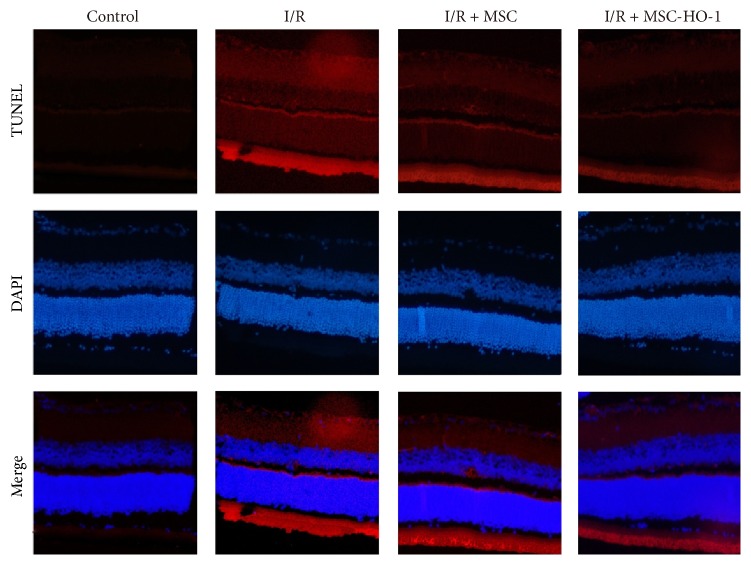
Effects of MSC-HO-1 on retinal cell apoptosis after I/R injury. At day 21, the retinas of control, I/R + PBS, I/R + MSC, or I/R + MSC-HO-1 group were stained utilizing TUNEL and DAPI. TUNEL staining is denoted in red and DAPI staining of nuclei in blue.

As showed in Figure 5, the fluorescent image of tissues clearly displayed that there were much more TUNEL-positive cells in the retinas of the I/R only group than the control group, whereas the amount of TUNEL-positive cells of retinas in animals receiving MSC transplantation was markedly reduced. Meanwhile, MSC-HO-1 further reduced TUNEL-positive cells in retina, indicating potent preservation against I/R-induced apoptosis.

### 3.6. Effects of MSC-HO-1 on the Expression of Retinal Apoptosis-Related Protein

**Figure 6:**
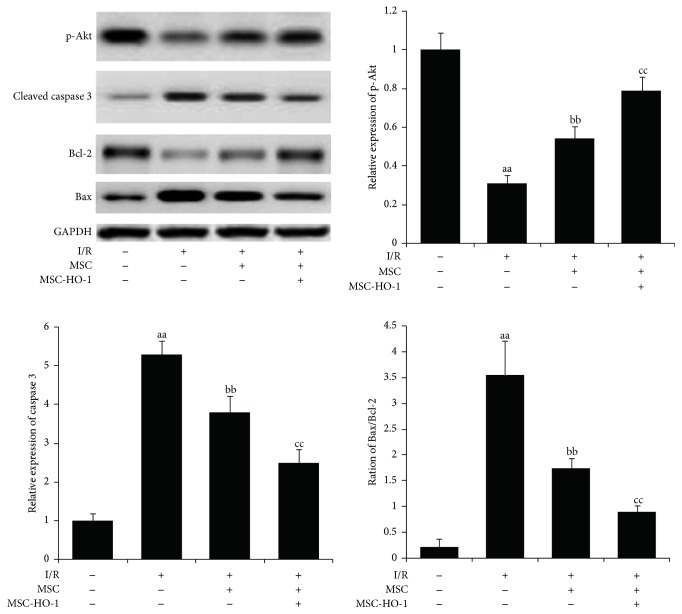
Effects of MSC-HO-1 on the expression of apoptosis-related proteins in retinal cells after I/R injury. At day 21, the expression of apoptosis-related proteins in retinas of control, I/R + PBS, I/R + MSC, or I/R + MSC-HO-1 group was investigated via western blotting (*n* = 5). ^aa^*P* < 0.01 compared with control group; ^bb^*P* < 0.01 compared with IR + PBS group; ^cc^*P* < 0.01 compared with IR + MSC group.

21 days after I/R injury, the expression of antiapoptosis factor p-Akt was significantly downregulated compared with the control (Figure 6). When introducing MSC, the expression of p-Akt was significantly promoted. This promotion of p-Akt expression in I/R injury retinas was further strengthen by the overexpression of HO-1 in MSC. On the contrary, I/R injury amplified the expression of proapoptosis factors, cleaved caspase 3 and Bax/Bcl-2 ratio, which could be significantly offset by MSC, especially MSC-HO-1.

### 3.7. Effects of MSC-HO-1 on the Level of ROS and the Activity of Antioxidases

**Figure 7:**
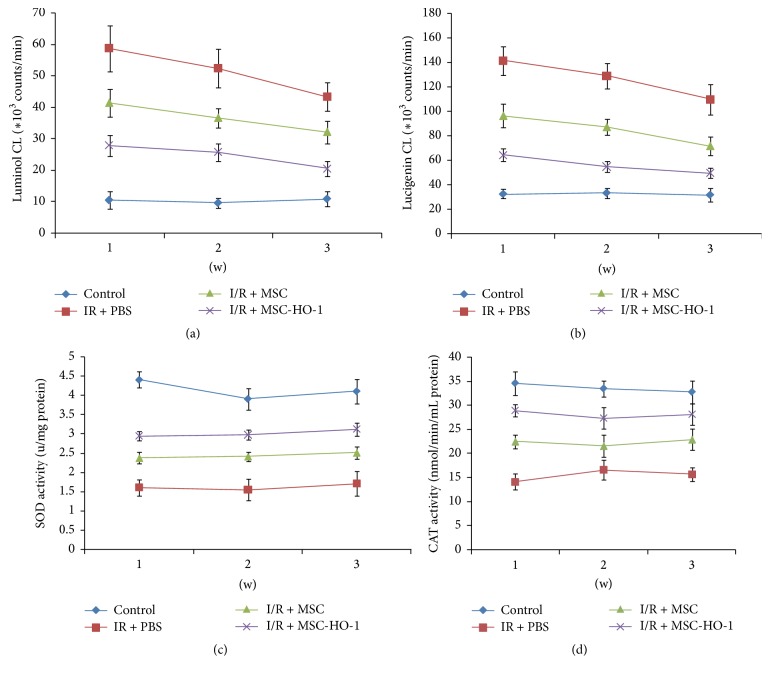
Effects of MSC-HO-1 transplantation on retinal ROS and antioxidase activity after I/R injury. (a) Luminol- and (b) lucigenin-enhanced chemiluminescence and the activity of (c) SOD and (d) CAT enzymes in retinas of the control, I/R + PBS, I/R + MSC, or I/R + MSC-HO-1 group were investigated at weeks 1, 2, and 3, respectively (*n* = 5).

We finally examined the changes of oxidative states in retinas by assessing the level of ROS and the activity of antioxidant enzymes. First, we employed lucigenin- and luminol-enhanced chemiluminescence (CL) methods to investigate the ROS level at weeks 1, 2, and 3, respectively. The counts of luminol- and lucigenin-enhanced CL could be dose-dependently quenched by ROS and superoxide. Administration with MSC significantly decreased mean luminol- and lucigenin-enhanced CL signal over 3 weeks compared with untreated group (Figures 7(a) and 7(b)). Similarly, HO-1 overexpression in MSC further reinforced the restoring effects. SOD and CAT played key roles in the antioxidant defense system. As shown in Figures 7(c) and 7(d), the significant decrease in the activity of SOD and CAT enzymes caused by retinal I/R injury was markedly suppressed by treatment with MSC, particularly MSC overexpressing HO-1 at weeks 1, 2, and 3.

### 3.8. Survival of MSC in Retina

**Figure 8:**
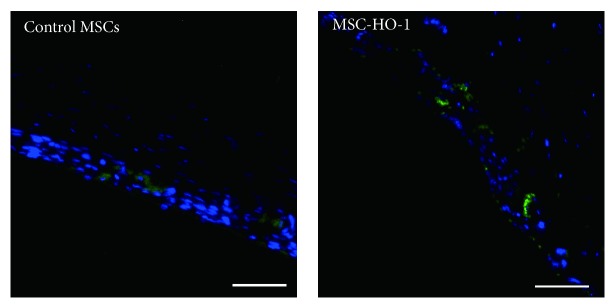
Survival of transplanted MSC in rat retina. After transplantation for 21 days, the survival of transplanted MSC was examined based on the expression of GFP. Bar = 50 *μ*m.

After transplantation for 21 days, we checked the viability of transplanted MSC in rat retina. As showed in Figure 8, in the control MSC, a few MSC was observed based on the expression of GFP. Meanwhile, much more MSC were identified in MSC-HO-1 group, indicating that HO-1 overexpression was also able to promote the survival of MSC, which may also strengthen the therapeutic effect of cell-based transplantation.

## 4. Discussion

The generation of ROS and thus oxidative stress has been regarded as one of main retinal damage induced by I/R injury [33, 34]. Thus, drugs or methods that are capable of attenuating ROS level in retina are the mainstream for retinal I/R injury treatments [33]. H_2_O_2_ belonged to one of the most important members in ROS, due to its relatively more stable and central role of ROS metabolism [35, 36]. These characteristics enable H_2_O_2_ gradually to become a model molecule for the in vitro study of ROS or oxidative stress [19, 37]. Here, we first employed H_2_O_2_ to simulate the in vitro oxidative stress microenvironment to investigate whether MSC have a protective effect on retinal cells, particularly retinal ganglion cells (RGC), which play a pivotal role to fulfil the physiological function of retina [38].

Conventionally, stem cells based transplantation therapies were considered as their capacity to differentiate into multiple cells after engrafting. However, this point of view has been challenged [39] because the therapeutic benefit could still sustain in vivo, even though the transplanted cells were vanished [40]. Thus, paracrine secretion roles of stem cells, reinforcing and improving function and structure of host cells and tissues, were gradually valued and studied broadly [16]. Utilizing an indirect coculture manner by transwell system, we found that MSC could significantly reduce intracellular ROS level and thus apoptosis in RGC-5. The next measurement of the apoptosis-related factors gave us more details about the MSC-based protection on RGC in molecular level. The augment of intracellular ROS blocked the Akt pathway [41], causing cell death by the inhibition of survival signals, such as Bcl-2 and the activation of proapoptotic signals, including Bax and cleaved caspase [42]. Meanwhile, the high level of ROS could also open the permeability transition gates of mitochondria, resulting in releasing apoptosis-activating proteins [43]. On the membrane outer layer of mitochondria, the expression of proapoptotic protein, Bax, was initiated, while the expression of antiapoptotic protein, Bcl-2, was inhibited. Further apoptotic response also stimulated the release of CytoC, ultimately activating the late-stage apoptotic protein, cleaved caspase 3 [44, 45]. Despite the plausible restoration of RGC, the therapeutic efficiency of MSC was far more from enough, especially compared with the normal RGC, which encouraged us to seek for an alternative strategy to improve MSC-based therapy of RGC.

In comparison with traditional drugs or nutrient based treatment, one of the most important benefits using cells therapy is that individual cell can serve as a “factory,” in situ producing certain factors. Owing to the development of gene engineering, cells could be modified by exogenous gene, specifically overexpressing certain factors or proteins [46, 47]. HO-1 has been evidenced to possess the antioxidative and protective properties, preventing toxic effect of oxidative stress on retinal endothelial cells. We thus incorporated MSC with HO-1 gene to obtain MSC overexpressing HO-1. The protection of H_2_O_2_-treated RGC by MSC-HO-1 indicated that the overexpression of HO-1 in MSC could greatly promote the therapeutic efficiency of MSC, increasing up to 2 times as that of MSC alone. Likewise, the oxidative stress induced expression of apoptosis-related proteins was largely offset. Together, these findings suggested that overexpression of HO-1 strengthened the therapeutic potential of MSC for retinal I/R injury.

Afterwards, we performed I/R injury in rat retina and observed that I/R injury caused the structural loss of retina, reaping flimsy tissues. The following apoptosis examination revealed that a myriad of cells suffered apoptosis, expressing more proapoptotic proteins and less antiapoptotic factors after I/R injury. Many studies have suggested that I/R injury would generate a large amount of ROS, resulting in cellular damage in varied animal models [1, 33]. We thus assessed the in vivo ROS level in a comprehensive way with luminol and lucigenin. The former one responded to a variety of radicals, including H_2_O_2_, OH^−^, hypochlorite, and peroxynitrite, while lucigenin served as the detector to superoxide radicals. Our data of in vivo ROS analysis were in accordance with previous studies [32], indicating that I/R injury induced apoptosis in retina was accompanied by the significant augments of ROS level. Additionally, the high level of ROS suggested the decrease of oxidant scavenges, typically SOD and CAT [32]. After I/R injury, the activities of antioxidant enzymes, SOD and CAT, in retina were dramatically hampered, further weakening the capacity of degrading intracellular ROS.

MSC were then implanted into rat retina and their therapeutic effects were evaluated. MSC were reported to be able to secrete a variety of tropic or antioxidative factors, which were conducive to restore adjacent microenvironment and cells. HO-1, as a potent antioxidant enzyme [37], further attenuated the increase of ROS level. After transplantation of MSC overexpressing HO-1, the abnormalities of both ROS level and representative antioxidant enzyme activities were dramatically corrected. The decrease of oxidative stress facilitated lowering the expression of apoptosis-related proteins in damaged retina. The proapoptotic proteins, cleaved caspase 3 and Bax, were strongly downregulated, while the antiapoptotic proteins, p-Akt and Bcl-2, were significantly upregulated. The decrease of retinal cells suffering apoptosis contributed to the recovery of retina, ultimately leading to the marked augment of retinal thickness. Albeit plausible results in retinal treatment after I/R injury, how the proteins or factors secreted from MSC affected surrounding cells was still obscure. We hence need more detailed investigations to verify related signal pathways in the future.

In conclusion, we here utilized the in vitro system of H_2_O_2_-simulated oxidative stress to imitate retinal cells suffering I/R injury, revealing that overexpression of HO-1 could enhance the protection of MSC on retinal cells. The next in vivo study demonstrated that I/R injury induced oxidative stress could be potently attenuated by transplanting MSC-HO-1. The reduction of apoptotic retinal cells and thus the raise of retinal thickness fully evidenced that the therapeutic effects of MSC on I/R injury were improved by HO-1 overexpression.
